# Layer‐Dependent Effect of Aβ‐Pathology on Cortical Microstructure With Ex Vivo Human Brain Diffusion MRI at 7 Tesla

**DOI:** 10.1002/hbm.70222

**Published:** 2025-05-02

**Authors:** Zhiyong Zhao, Zuozhen Cao, Qinfeng Zhu, Haoan Xu, Sihui Li, Liangying Zhu, Guojun Xu, Keqing Zhu, Jing Zhang, Dan Wu

**Affiliations:** ^1^ Children's Hospital Zhejiang University School of Medicine, National Clinical Research Center for Child Health Hangzhou China; ^2^ Key Laboratory for Biomedical Engineering of Ministry of Education, Department of Biomedical Engineering College of Biomedical Engineering & Instrument Science, Zhejiang University Hangzhou China; ^3^ National Human Brain Bank for Health and Disease Zhejiang University Hangzhou China; ^4^ Department of Pathology The First Affiliated Hospital and School of Medicine, Zhejiang University Hangzhou China

**Keywords:** Alzheimer's disease, Aβ‐pathology, cortical layer, ex vivo MRI, microstructure

## Abstract

The laminar‐specific distributions of Aβ and Tau deposition in the neocortex of Alzheimer's disease (AD) have been established. However, direct evidence about the effect of AD pathology on cortical microstructure is lacking in human studies. We performed high‐resolution T2‐weighted and diffusion‐weighted MRI (dMRI) on 15 ex vivo whole‐hemisphere specimens, including eight cases with low AD neuropathologic change, three cases with primary age‐related tauopathy (PART), and four healthy controls (HCs). Using the diffusion tensor model, we evaluated microstructure patterns in six layers of gray matter cortex and performed MRI‐histology correlation analysis across cortical layers. Aβ‐positive cases exhibited higher diffusivity than Aβ‐negative cases (PART and HC) in selected cortical regions, particularly in the inferior frontal cortex. Both Aβ/Tau depositions and dMRI‐based microstructural markers demonstrated distinct cortical layer‐dependent and region‐specific patterns. A significant positive correlation was observed between increased diffusivity and Aβ burden across six cortical layers but not with Tau burden. Furthermore, the mean diffusivity in layer V of the inferior frontal cortex significantly increased with the Amyloid stage. Our findings demonstrate a layer‐dependent effect of Aβ pathology on cortical microstructure of the human brain, which may be used to serve as a marker of low AD neuropathologic change.

## Graphical abstract

Aβ‐pathology has a layer‐dependent effect on dMRI‐based microstructures of the neocortex, which may serve as a marker of early AD neuropathologic change.

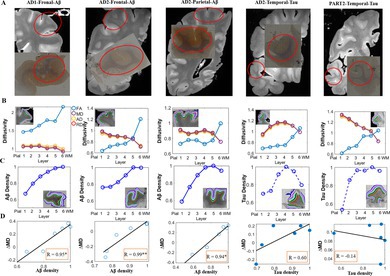

## 1 Introduction

The human neocortex consists of six cytoarchitectonic cortical layers, each characterized by distinct cell density, size, and type. These layers are connected to different brain areas (Tomer et al. 2022; Von Economo and Koskinas 1925) and exhibit specific gene expression patterns (James et al. 2022; McColgan et al. 2021). Alzheimer's disease (AD) is characterized by widespread cellular degeneration and the presence of tau intracellular neurofibrillary tangles (NFTs) and extracellular amyloid‐β (Αβ) plaques. The distribution of Aβ burden in the neocortex of AD brains is layer‐specific, with deep layers in the prefrontal (Majocha et al. 1988) and temporal cortex (Delaere et al. 1991) being particularly affected. Tau‐induced NFTs selectively affect different cell types in different regions, for example, pyramidal neurons in the neocortex (Otero‐Garcia et al. 2020) and stellate and multipolar neurons in the entorhinal cortex (Lee, et al. 2021). Therefore, the effect of AD pathology on neocortical microstructure may vary across different cortical layers.

Diffusion MRI (dMRI) provides a non‐invasive way to probe tissue microstructures in both white matter (WM) and gray matter (GM) and their pathological changes in brain disorders (Lee et al. 2020; Sampedro et al. 2019; Stock et al. 2020). From a histopathological perspective, AD primarily affects the cortex, especially in its early stages (Braak and Braak 1998). Thus, the use of dMRI to detect microscopic cortical GM abnormalities, such as diffusivity changes, may be a powerful tool for identifying early AD changes (Weston et al. 2015). However, the relationship between these microstructural markers and AD pathology in the human cortex is not yet known, which demands MRI and pathology of the same brain.

The thickness of each of the six cytoarchitectonic layers in the human GM cortex ranges from approximately 0.2 mm to 0.8 mm (Wagstyl et al. 2020). Due to limited spatial resolution (> 1 mm), in vivo dMRI is unable to distinguish six cortical layers but typically divides the GM cortex with a thickness of 1–4.5 mm (Fischl and Dale 2000) into three depths: superficial, middle, and deep. These studies have revealed depth‐dependent microstructural patterns in the cortex (Ali et al. 2022; Bletsch et al. 2021) and structural abnormalities in several diseases, including autism spectrum disorder (Bletsch et al. 2021), schizophrenia (Wei et al. 2022), and bipolar disorder (Suh et al. 2023). A recent in vivo dMRI study with 0.9 mm isotropic resolution used a column‐based cortical depth analysis to observe local maximum/minimum values of fractional anisotropy (FA) and maximum radiality index at intermediate cortical depths in most regions (Ma et al. 2023). Although in vivo dMRI studies have revealed microstructural changes in the cortex of AD brain, such as decreased radiality in the frontotemporal cortex (Lee et al. 2020) and increased mean diffusivity (MD) in the temporoparietal areas (Montal et al. 2018), it remains unclear whether these changes are layer‐dependent within the cortical region.

Compared to in vivo MRI, ex vivo scans allow for higher resolutions given the unlimited scan time, enabling clear anatomical delineation of the cortical layer. FA/MD with local maxima and minima at specific cortical depths have been observed in ex vivo primary visual cortex (Kleinnijenhuis et al. 2013), primary sensorimotor cortex (Balasubramanian et al. 2021), and prefrontal cortex (Aggarwal et al. 2015). More importantly, ex vivo MRI enables direct MRI‐histology correlation of the same human brain. Our recent studies found layer‐specific microstructural changes in the hippocampus that significantly correlated with Aβ/Tau protein content in AD samples (Zhao et al. 2022). However, the aforementioned ex vivo dMRI studies all focused on specific small tissue blocks and did not characterize how layer‐specific Aβ/Tau deposition affects the cortical microstructure across the whole hemisphere.

It is well known that AD is a progressive neurodegenerative disorder, and its early diagnosis and subsequent access to the appropriate treatments can help the patients gain control of the disease and improve their quality of life. The pathological changes in the AD brain have already occurred at least 10–20 years earlier than the onset of typical clinical symptoms (Long and Holtzman 2019). Therefore, it is critical to study brain changes in the early stages of AD pathology for understanding its development progress. Primary age‐related tauopathy (PART) has similar features in neuronal tau deposits with AD (Duyckaerts et al. 2015), but these patients have different cognitive outcomes and overall morbidity (Bell et al. 2019; Teylan et al. 2019). Whether PART pathology inevitably progresses to AD has been controversial (Jellinger et al. 2015). Here, we treated the PART as an intermediate stage between healthy controls (HC) and AD. In this study, we collected submillimeter‐resolution dMRI images from 15 ex vivo whole hemispheres, including four HC, three PART, and eight cases with low AD neuropathologic change (ADNC) (Hyman et al. 2012), aiming to (1) identify cortical microstructure as a function of cortical layer in the GM cortex; (2) compare differences in layer‐specific dMRI measurements between groups; and (3) characterize the relationship between dMRI metrics and Aβ/Tau density across cortical layers in the early stages of AD pathology. We hypothesized that Aβ/Tau pathology would have a layer‐dependent effect on cortical microstructure.

## 2 Materials and Methods

### 2.1 Sample Preparation

Fifteen fresh brains (Table 1) were dissected within 8 h of death according to the Standardized Operational Protocol for Human Brain Bank in China (Qiu et al. 2018). All tissues were obtained with donor consent and provided by the National Health and Disease Human Brain Tissue Resource Center (http://zjubrainbank.zju.edu.cn/index). Each right hemisphere was fixed in 6% paraformaldehyde (PFA) for 28 days and was transferred to phosphate‐buffered saline (PBS) for 2 days (Hade et al. 2024) to wash out fixative, which has been reported to raise the apparent diffusion coefficient (ADC) by approximately 30% (D'Arceuil et al. 2007). The specimens were then placed in a homemade container filled with Fomblin and stabilized with supporting materials during the MRI scan. AD and PART diagnoses were confirmed at autopsy, and controls were selected from cases with no vascular or other neurological complications. None of the AD participants met the criteria for intermediate or high ADNC (Hyman et al. 2012). Moreover, while abnormalities in vascular structure significantly influence diffusional measures (Badji and Westman 2020; Finsterwalder et al. 2020), we cannot assess the vascular alteration for each case because the vessels collapsed in the ex vivo sample. Ethical approval for all experimental procedures was obtained from the ethics committee of Zhejiang University School of Medicine.

**TABLE 1:** Summary of human brain hemisphere samples.

Sample	Age	Sex	Brain weight(g)	Hemisphere	PMI (min)	FT (day)	ABC score	Braak stages	Amyloid stages	Vascular pathology	TDP‐43
HC1	58	M	1396	Right	1075	77	A0B0C0	0	0	−	−
HC2	64	M	1279	Right	372	32	A0B0C0	0	0	−	−
HC3	54	M	1429	Right	298	42	A0B0C0	0	0	−	−
HC4	48	F	1162	Right	669	36	A0B0C0	0	0	−	−
PART1	89	M	1249	Right	885	40	A0B1C0	1	0	−	+
PART2	74	M	1230	Right	161	39	A0B1C0	2	0	−	+
PART3	63	M	1065	Right	967	58	A0B2C0	4	0	−	−
AD1^a^	54	M	1312	Right	351	38	A1B0C0	0	1	−	−
ad2^a^	66	M	1377	Right	660	45	A1B0C0	0	1	−	−
AD3^a^	65	M	1295	Right	469	56	A1B1C0	1	1	−	−
ad4^a^	80	M	1171	Right	353	35	A1B1C0	2	1	−	+
a5^a^	73	M	1043	Right	264	31	A1B2C0	3	2	−	+
ad6^a^	70	M	1202	Right	294	32	A1B1C0	2	2	CAA1	+
a7^a^	73	M	1272	Right	331	40	A1B2C0	3	2	−	+
ad8^a^	80	M	1224	Right	702	32	A1B1C0	1	1	−	+

*Note:* There were no significant differences observed between Aβ‐negative and Aβ‐positive groups (PMI: *p* = 0.17; FT: *p* = 0.25).

Abbreviations: CAA, cerebral amyloid angiopathy; FT, fixation time; NA, not available; PMI, postmortem interval; TDP‐43, TAR DNA‐binding protein of 43 kDa.

^a^ These cases are Aβ‐positive (termed AD for simplicity) but do not meet the diagnostic criteria for AD. Braak stage is defined based on neurofibrillary changes: transentorhinal stages I–II, clinically silent cases; limbic stages III–IV, incipient AD; neocortical stages V–VI, fully developed AD. Amyloid stage is defined based on amyloid accumulation: starting from the neocortex (phase I), progressing to the allocortex in phase II (hippocampus, entorhinal cortex and cingulate gyrus), and extending to the basal ganglia (phase III), midbrain (phase IV) and cerebellum (phase V) in the later stages.

### 2.2 MRI Acquisition

All MRI scans were performed on a Siemens 7T MAGNETOM scanner with a 32‐channel head coil. T2‐weighted images were obtained with the following parameters: repetition time (TR) = 12,510 ms; echo times (TE) = 24 ms; voxel size = 0.5 × 0.5 × 0.6 mm^3^; and 10 averages. dMRI was acquired with a 3D diffusion‐weighted steady state free precession (DW‐SSFP) (Bieri et al. 2012) sequence with the following parameters: TR = 29 ms; TE = 21 ms; voxel size = 0.8 × 0.8 × 0.8 mm^3^; 20 images (b0) without diffusion weighting and 60 non‐colinear diffusion directions at a b‐value of 6000 s/mm^2^.

### 2.3 Immunohistochemistry Staining

Following MRI scans, immunohistochemistry was performed on formalin‐fixed, paraffin‐embedded tissues for autopsy. Brain blocks were dissected from the same brains and then fixed in 4% PFA in 0.1 M PBS (pH 7.4) for 2 days before being cryopreserved in 15% sucrose in 0.01 M PBS (pH 7.4). Blocks were then sectioned at a thickness of 3 μm using a microtome. The immunohistochemistry staining protocol and information on all antibodies used in this study are provided in Table S1.

Histological images used in this study included four H&E staining images and four Klüver–Barrera staining images obtained from four cortical regions of one sample, and five pathological staining images from three cortical regions of three samples (Table 2). Note that staining was performed for all disease samples, but only the images with sufficient anatomical information were selected for quantitative analysis, while those with inadequate anatomical information or severe tissue damage (difficult to be matched to MRI) were excluded. We have maximized the utility of all available samples.

**TABLE 2:** Samples information of histological images.

Sample	Histological staining	Brain regions
PART1	H&E and Klüver‐Barrera staining	Middle frontal gyrus^a^, superior parietal lobe^a^, temporal pole^a^ and occipital pole^a^
PART3	Tau staining	Temporal pole
AD1	Aβ staining	Middle frontal gyrus
AD3	Aβ staining	Middle frontal gyrus, superior parietal lobe
AD3	Tau staining	Temporal pole

^a^ Two slices were extracted in each block to perform H&E and Klüver–Barrera staining, respectively.

### 2.4 Cortical Layer Segmentation

**FIGURE 1:**
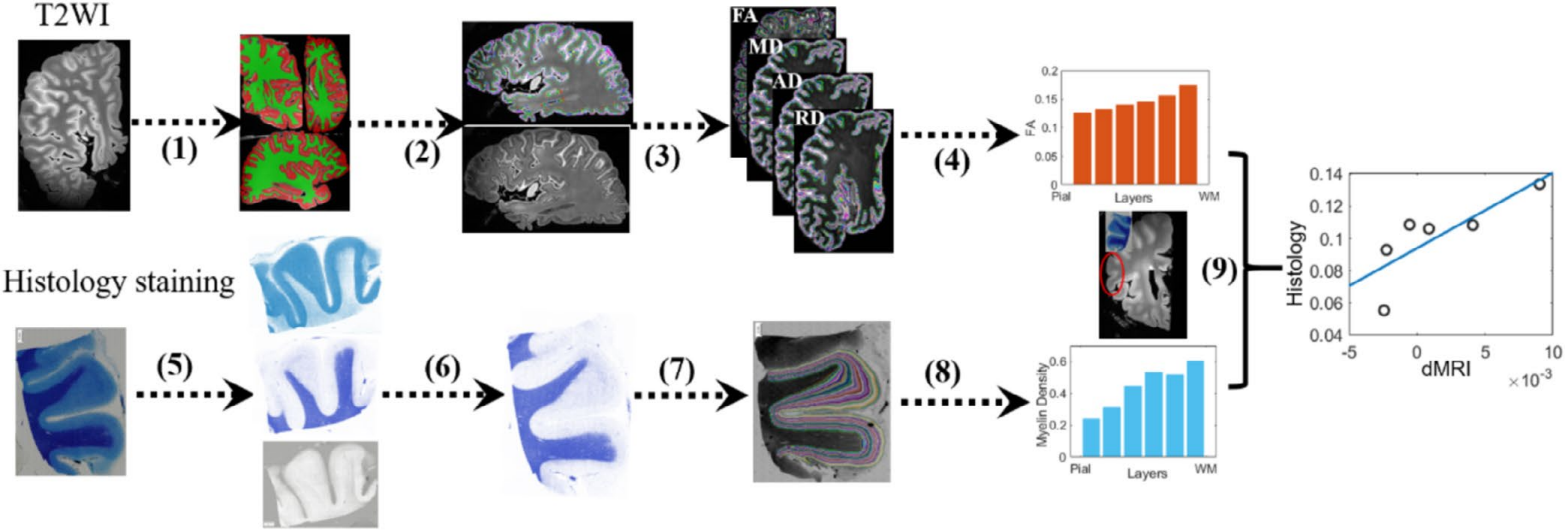
The pipeline of data processing. (1) and (2) represent segmentation of GM/WM and cortical layers in T2‐weighted image, respectively; (3) represents the calculation of diffusion measures; (4) shows averaged values of each dMRI metric in each layer; (5–8) represent the color deconvolution, Moment thresholding, laminar segmentation and averaged values of histological measures at each layer, respectively; (9) shows the correlations between dMRI and histology. AD, axial diffusivity; FA, fractional anisotropy; MD, mean diffusivity; RD, radial diffusivity.

The automated segmentations of ex vivo brain MRI based on machine learning have been reported in recent studies (Zeng et al. 2023; Khandelwal et al. 2024; Casamitjana et al. 2024). As depicted in Figure 1, the whole hemisphere was segmented into GM and WM based on the T2‐weighted image (T2WI) using the nnU‐net algorithm (Isensee et al. 2021) in this study. Specifically, for each ex vivo hemisphere, in the same locations, we manually labeled GM/WM in whole slide on five slices per brain with a 3 mm interval between adjacent slices along the sagittal axis. The subcortical GM and white matter hyperintensities were not labeled separately on each slice, and they were labeled as a part of GM and WM, respectively. Then, 50 slices of 10 of 15 hemispheres (four Aβ‐negative cases and six Aβ‐positive cases) were used as the training dataset, and the 25 slices of the remaining five samples were used as the test dataset. The 2D U‐Net was trained using PyTorch 1.8.1 and Nvidia GeForce RTX3090 with a batch size of 8, a patch size of 448 × 256, a depth of 7, and a base channel number of 32. Notably, after training nnU‐net by using manual segmentations, we manually corrected nnU‐net segmentations and used them to retrain the nnU‐net. The model was optimized using a loss function based on the Dice coefficient (DC) and cross‐entropy, and fivefold cross‐validation was employed during training. Test results in the remaining five samples showed high prediction accuracy with an average DC > 0.8. The trained model was then used to predict the segmentation of whole hemispheres for each sample. More details related to the nnU‐net method were displayed in Supporting Information. Following auto‐segmentation, we visually inspected the GM/WM labels slice by slice across axial, sagittal, and coronal views, and manually corrected the possible segmentation errors in slices with little tissue (Figure S1) or regions with boundary ambiguities (Figure S2) or areas with buried sulci (Figure S3). Moreover, we randomly selected five slices and manually segmented GM‐WM along the axial and coronal axis, respectively, in an Aβ‐positive case (ad3) and an Aβ‐negative case (PART1), and then calculated their DC with the GM‐WM segmentations from the 2D nnU‐net model trained on sagittal slices. The results showed high DC with an average value of 0.83 for GM and 0.82 for WM (Figure S4C), suggesting that the model is potentially effective for GM‐WM segmentation in axial and coronal slices. In marginal areas exhibiting very low signal due to coil sensitivity, GM‐WM segmentation was omitted, and these areas were excluded from the final analysis.

**FIGURE 2:**
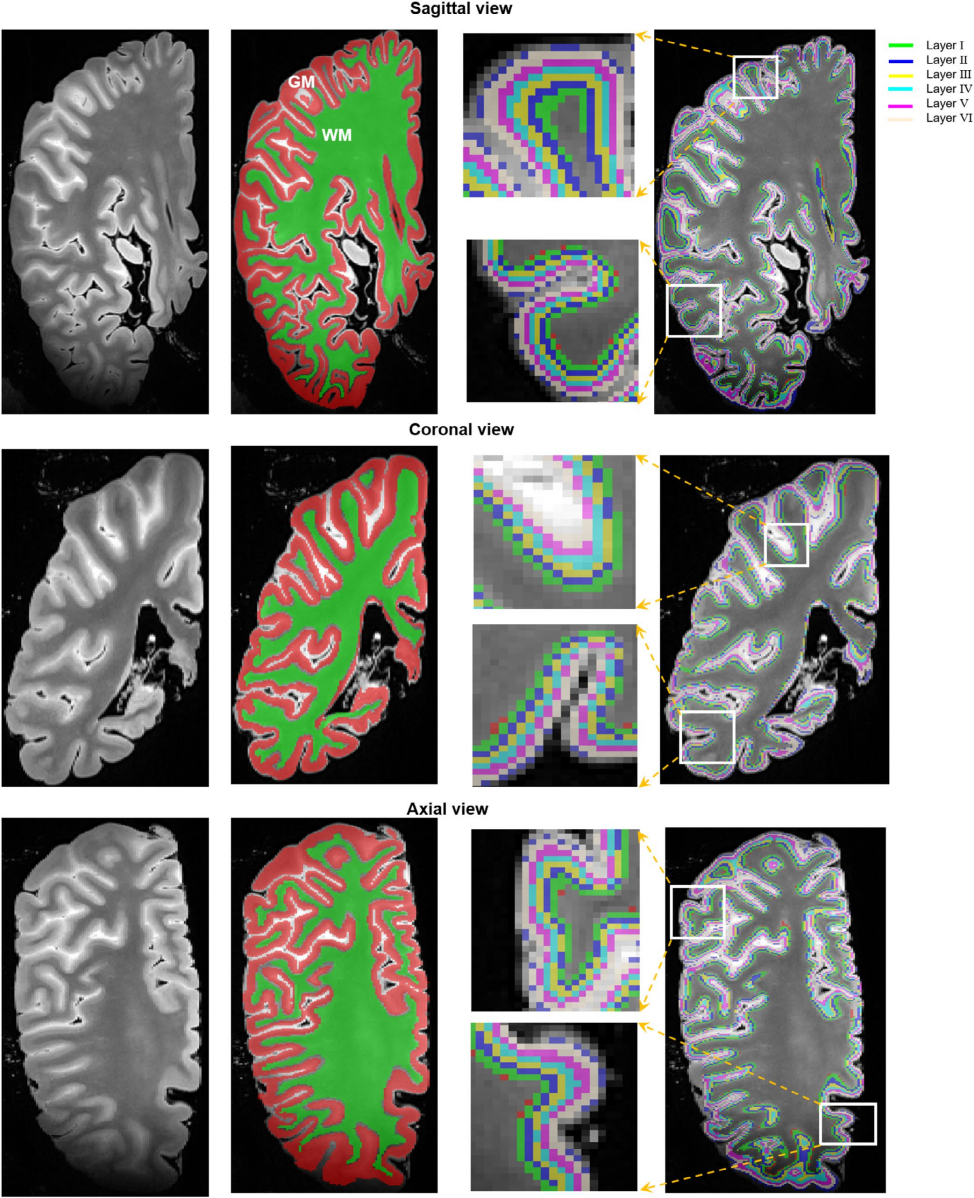
The display of GM‐WM and cortical layer segmentations in three slices of one case across sagittal, coronal, and axial views, respectively. The six cortical layers of GM cortex are shown, excluding the innermost and outermost laminae from eight equidistant laminae.

The boundaries of GM‐CSF (pial surface) and GM‐WM (WM surface) were obtained by dilating the GM and WM masks by one voxel using FSL (https://fsl.fmrib.ox.ac.uk/fsl/fslwiki/) and intersecting the dilated images with the original GM mask. The pial surface, WM surface, and GM mask were then input into the LAYNII tools (Huber et al. 2021) to divide the entire GM cortex into eight equidistant laminae for each case. The innermost and outermost laminae were excluded to reduce segmentation errors due to partial volume effects. The GM‐WM and cortical layer segmentations in three slices of one case across sagittal, coronal, and axial views are shown in Figure 2. Then, the cerebral cortex of the ex vivo hemispheres was parcellated into 180 parcels based on the HCP atlas (Glasser et al. 2016), after coregistration between the HCP atlas (right hemisphere) to the T2‐weighted ex vivo images using a nonlinear method within the GM mask from nnU‐net segmentation in ANTs (http://stnava.github.io/ANTs/). Here, our coregistration process included several critical steps: Initially, we masked out the CSF from the in vivo template in MNI space before registration to enhance anatomical similarity with the ex vivo MR images. Subsequently, a two‐channel approach utilizing T2‐weighted intensity images and GM/WM labels was used to nonlinearly register the ex vivo images to the in vivo template space for each subject. This process yielded an inversion transformation, which was then applied to the HCP atlas to derive individualized images. Next, the transformed GM label was masked by the individual GM mask obtained from nnU‐net segmentation. Then, we assessed registration accuracy using thickness similarity (Zhang et al. 2022), defining parcels with a thickness difference of < 0.5 mm (equivalent to one voxel of T2‐weighted MR used in our study) compared to HCP reference data (Glasser et al. 2016) as targeted regions‐of‐interest (ROIs). Finally, 28 ROIs passed the evaluation in all samples and were selected for the following analysis. Each ROI was multiplied by the layer segmentation, and the final labels were transferred from T2‐weighted images to dMRI data. The reliability of segmentation was also examined by comparing its consistency among four HC samples in Figure S5.

### 2.5 Processing of the dMRI and Histological Data

FA, MD, axial, and radial diffusivity maps were calculated using the tensor model from the dMRI data after denoising and bias field correction in MRtrix3 (https://www.mrtrix.org/) and then were normalized with respect to the mean value of the entire cortex. These quantitative maps were registered to T2‐weighted space using linear registration in FSL. Finally, averaged values of the four normalized dMRI measures (e.g., mFA and mMD) were extracted for each layer in ROIs for each sample.

For histological images, the fractions of cells, myelin, Aβ, and Tau in each layer were quantified in H&E, Klüver‐Barrera, Aβ, and AT8 staining, respectively, using Image‐J (https://imagej.net/). Notably, according to a previous review (Lazari and Lipp 2021) that highlighted Luxol Fast Blue (LFB) as the most frequently used technique in MR‐histology validation for myelin, the LFB is attracted by the basic amino acids of myelin proteins and by phospholipids (e.g., Klüver‐Barrera staining (Klüver and Barrera 1953; Morgan et al. 2021)). Taking H&E staining as an example, we initially performed segmentations where stained images underwent color deconvolution (Ruifrok and Johnston 2001) using a manually chosen vector with the “From ROI” option. The resulting channel 3 output was then extracted as it was mostly represented by cell staining with minimal background contribution. This approach has been validated in prior studies (Bagnato et al. 2018; Zhou et al. 2020). Subsequently, the cell staining was locally thresholded using the Moment‐preserving threshold (Tsai 1985) method. The degree of cell staining was determined by the proportion of image‐covered staining (signals above the threshold levels) relative to each brain slice (Zhou et al. 2020). Furthermore, boundaries of CSF‐GM and WM‐GM were manually delineated for each histological image, and laminar segmentation was performed using the same approach as for T2‐weighted images. Finally, the ratio of positive pixels to total pixels within each layer on thresholded images was defined as the cell density in that layer.

### 2.6 DMRI‐Histology Correlation Analysis

Note that in this study, 2D histological images were obtained from several small tissue blocks, while 3D MRI covered the entire hemisphere. Direct coregistration between MR and histological images was challenging, due to the vast difference in terms of field of view, data format, as well as the distortion in histological samples. Instead, a qualitative registration was performed by aligning corresponding areas within the same brain based on prior anatomical knowledge in the current study. Our aim is not voxel‐wise correlation but rather the correlation between the layer‐dependent microstructural profiles. Therefore, we consider this approximate alignment acceptable, provided the MR and histological images originate from the same anatomical location. The tissue blocks used in the current study were dissected from the whole brain according to a common anatomical segmentation protocol and 2D slices used for histological staining were extracted from the same tissue along the coronal axis. The most similar anatomical region to the histology image in the 3D MRI was visually identified by a neuroanatomist with 30 years of experience (Z.K.) and an MR physicist (Z.Z.) with 11 years of experience.

### 2.7 Statistical Analysis

The main data analysis included the following steps. (1) Considering the significant difference between Aβ‐negative and Aβ‐positive groups in averaged cortical thickness (two‐sample *t*‐test: *p* = 0.001, *t* = −4.12), and the correlations between dMRI measures and PMI as well as FT (PMI: *p* = 0.17; FT: *p* = 0.25, Figure S6), we compared the differences of FA/MD between two groups in each of six layers in 28 parcels using a two‐sample *t*‐test after regressing out covariates such as age, sex, cortical thickness, PMI, and FT. 2) The relationship between dMRI measurements and age/Braak stage/Amyloid stage was evaluated using a linear mixed model (y–1 + Age + Sex + Braak stage + Amyloid stage + (1 | Subject)) in each cortical layer of each ROI. 3) The changes in cell/myelin density and Aβ/Tau protein burden in cortical layer were analyzed and correlated with dMRI measurements across six layers using Spearman correlation. Notably, when performing correlation analysis with Aβ/Tau density, we used changes in dMRI measurements (e.g., ΔMD) between the PART or AD and its age and sex‐matched HC cases, and this method was used in previous disease study (Romme et al. 2017).

## 3 Results

### 3.1 Cortical Layer‐Specific and Region‐Specific Patterns of dMRI‐Based Microstructures

**FIGURE 3:**
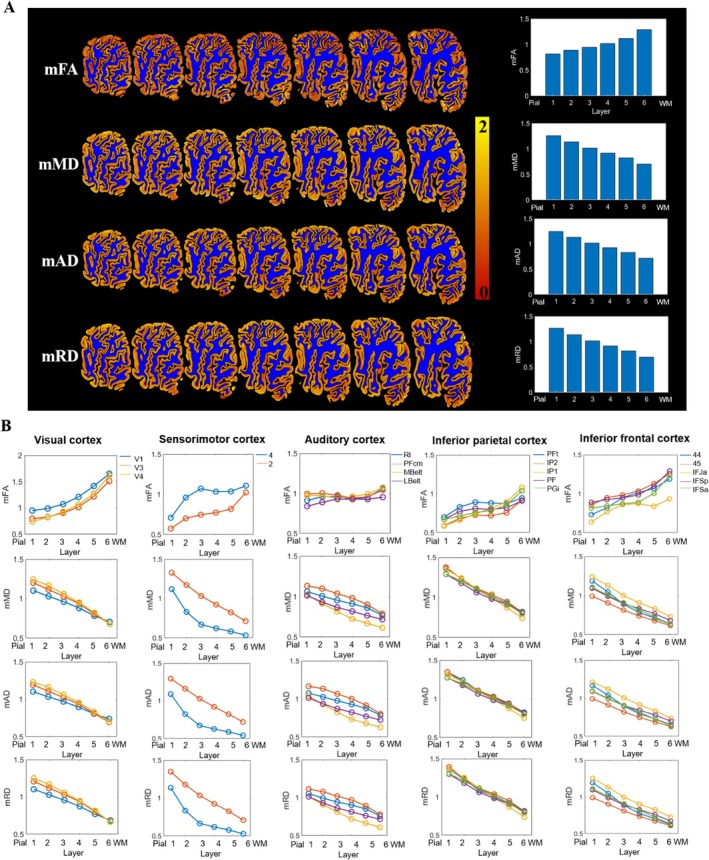
(A) Microstructural maps of the neocortex of the entire hemisphere and averaged values in each cortical layer and (B) layer‐dependent changes of dMRI measurements in different parcels (with different color lines) of one HC sample. From the pial to WM surface, FA and diffusivity show a gradual increase and decrease, respectively. Note that similar patterns were also observed in the other three HC samples, which are shown in the Supporting Information. The mFA, mMD, mAD, and mRD represent FA, MD, AD (axial diffusivity), and RD values in each parcel divided by the mean value of the entire cortex to minimize the individual differences.

Figure 3A displays maps of four dMRI measures in the neocortex and their averaged values in each cortical layer of an HC brain. FA increased, and diffusivity decreased from the pial to the WM surface. Additionally, layer‐dependent profiles differed among regions, as shown in Figure 3B for this brain specimen. Specifically, diffusivity decreased almost linearly with cortical layer in all cortical regions, while FA showed a possibly nonlinear increase that differed among regions, for example, the visual cortex and inferior frontal cortex showed the most layer‐dependent increase, followed by the inferior parietal cortex and sensorimotor cortex, and the auditory cortex had similar FA values across six layers. These patterns of anisotropy and diffusivity changes were consistently observed in four HC samples (Figures S7 and S8). Furthermore, cell and myelin density obtained from H&E and Klüver‐Barrera staining also displayed a layer‐dependent increase from the pial surface to WM in four cortical regions. After manual matching of MR and histological images, MRI‐histology correlation analysis revealed positive correlations between FA values and myelin density in the frontal and parietal lobes and negative correlations between MD values and cell density in the parietal lobe and occipital pole (Figure S9).

### 3.2 Cortical Layer‐Dependent Microstructural Changes in Aβ‐Positive Cases

**FIGURE 4:**
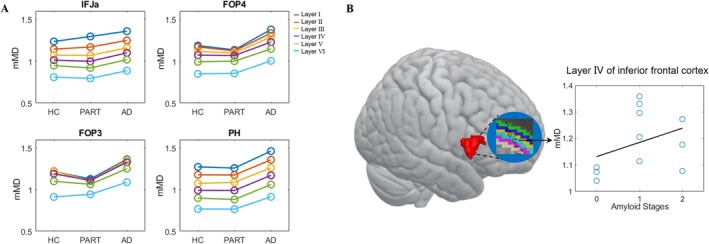
(A) Comparisons of layer‐dependent and region‐specific MD values among three groups in several ROIs and (B) a positive correlation between MD in layer IV of the opercular areas of the inferior frontal cortex and Amyloid stage. In the B panel, each dot represents a PART or AD subject, and the mMD label of the y‐axis represents the MD value in each parcel divided by the mean value of the entire cortex. IFJa, a subdivision of the inferior frontal sulcus; PH, parahippocampal areas; FOP3/4, frontal opercular area 3/4.

The layer‐dependent differences in MD among HC, PART, and Aβ‐positive groups were observed in many cortical regions, particularly in the inferior frontal cortex (IFC) and parahippocampal gyrus (PH) (Figure 4A). From HC to PART to Aβ‐positive group, MD values in the IFC and PH showed a gradually increasing trend, especially in deep layers, and such changes were not observed in other regions. Overall, Aβ‐positive group showed the highest MD among the three groups in six layers.

After evaluating registration accuracy, 28 out of 180 regions were included in subsequent statistical analyses. We conducted a total of 168 (28 brain regions × 6 cortical layers) group comparisons and correlation analyses, respectively. These statistical results were adjusted using false discovery rate (FDR) correction (*p* < 0.05).

We found that, after controlling for covariates of age, sex, cortical thickness, PMI, and FT, Aβ‐positive cases tended to exhibit higher MD values compared to Aβ‐negative cases in all layers (*p* < 0.05 for layers II/III and marginally significant in layer IV/V/VI due to the small sample size) (Table 3). No significant difference was found in FA. The ROI‐based results were not statistically significant and were not displayed here.

**TABLE 3:** Comparisons of FA/MD values between Aβ‐positive and Aβ‐negative groups in six cortical layers.

	Layer I	Layer II	Layer III	Layer IV	Layer V	Layer VI
FA						
*p*‐value	0.342	0.123	0.098	0.118	0.177	0.281
*T*‐value	1.010	1.724	1.876	1.754	1.482	1.157
MD						
*p*‐value	0.147	0.049*	0.048*	0.052	0.052	0.055
*T*‐value	−1.606	−2.311*	−2.350*	−2.281	−2.277	−2.245

*Note:* The *T* values presented in the table represent the results of a two‐sample *t*‐test conducted between Aβ‐positive and Aβ‐negative groups across brain regions in each cortical layer. Positive and negative *T* values indicate higher and lower FA/MD values in the former sample compared to the latter, respectively. *0.01 < *p* < 0.05.

The MD value in layer IV of the opercular areas of the IFC showed a significant positive correlation with Amyloid stage (*t* = 7.86, *p* = 1.01 × 10^−4^, adjusted *p* = 0.02) but not with age (Figure 4B). Layers I (*t* = 3.27, *p* = 0.01, adjusted *p* = 0.08), II (*t* = 4.18, *p* = 0.004, adjusted *p* = 0.07), and III (*t* = 4.41, *p* = 0.003, adjusted *p* = 0.07) also showed significant correlations but did not survive FDR correction.

We also did not observe significant differences between groups in FA/MD, significant correlations between Braak stage and FA/MD values, and between FA values and Amyloid stage or age after FDR corrections (*p* < 0.05).

### 3.3 Correlation Between dMRI‐Based Microstructures and AD Pathology

**FIGURE 5:**
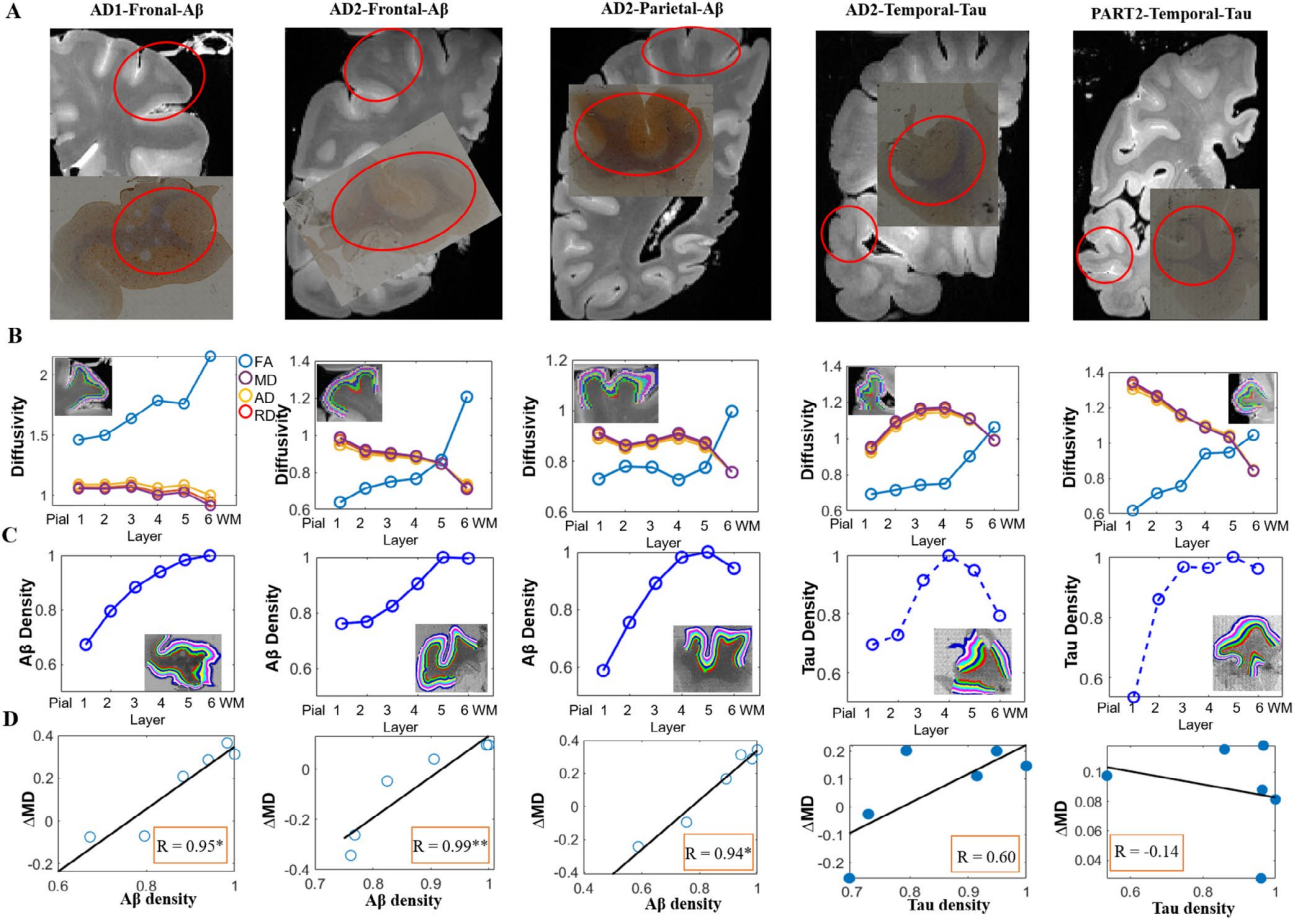
The relationship between dMRI measurements and Aβ/Tau density across six cortical layers in two Aβ‐positive (ad1/3) and one PART (PART3) samples. (A) Shows manual matching of MR sections with histological images; regions within red circles in the MRI are most similar to histological images. (B, C) display changes in dMRI indices and Aβ/Tau density with cortical layers from the pial surface to WM, respectively. (D) Shows correlations between ΔMD (difference between the MD in a layer in the patient and corresponding MD in that layer in the matched HC; paired cases: ad1‐HC3, ad3‐HC2 and PART3‐HC2) and Aβ/Tau density across six cortical layers. Here we hypothesized that the dMRI changes in patients, compared to normal controls, reflect microstructural impairments attributable to pathological protein burdens.

Quantitative protein density maps obtained from Aβ/Tau staining clearly showed plaque locations (indicated by red arrows in Figure S10) consistent with those observed in original staining images, which were aligned to corresponding MR images (Figure 5A). After being divided into six equidistant laminae, changes in dMRI measures and Aβ/Tau across six layers are shown in Figure 5B,C, respectively. Specifically, from the pial to the WM surface, the FA exhibited a nonlinear increase, consistent with patterns observed in HC cases. However, the diffusivity demonstrated a nonlinear decrease, in contrast to the linear decrease observed in HC cases. For instance, the diffusivity in the temporal lobe of ad3 displayed a “∩” change from the pial to the WM surface, peaking at layer IV (Figure 5B). For histological measures, Aβ deposition showed a nonlinear increase across cortical layers in the frontal cortex of ad1 (Aβ+ and Tau‐) and ad3 (Aβ+ and Tau‐) (Figure 5C). Tau burden displayed different patterns in the temporal cortex of PART3 (Aβ‐ and Tau+) and ad3 (Aβ‐ and Tau+), that is, Tau protein increased in layers I–III of PART3 and then remained unchanged in layers IV–VI, while it showed a “∩” change from the pial to the WM surface in ad3, peaking at layer IV. Correlation analysis revealed that changes in MD values (ΔMD, AD‐HC between paired samples) showed significant positive correlations with Aβ density in the frontal cortex of ad1/3 and parietal cortex of ad3 across six cortical layers (adjusted *p* < 0.05, FDR correction) (Figure 5D). No significant correlations were found between Tau density and MD values or between Aβ/Tau density and FA values. Note that we used ΔMD to remove the innate layer‐specific change but focus on AD‐related alterations.

## 4 Discussion

This study directly integrated high‐resolution dMRI and histological staining to investigate the relationship between cortical microstructure and AD pathology in ex vivo human brains. We found that cortical microstructure exhibited a layer‐dependent and region‐specific pattern in which diffusional anisotropy increased and diffusivity decreased in most cortical regions from the pial surface to WM. Aβ‐positive cases displayed higher diffusivity than other groups in specific layers in selected regions. Furthermore, diffusivity showed significant positive correlations with Aβ but not with Tau deposition across six cortical layers. These findings suggest that changes in layer microstructure within specific brain regions in cases with early AD pathology may potentially be linked to Aβ deposition across cortical layers.

### 4.1 Layer‐Dependent Microstructure in Cortical Regions

From the pial surface to WM, we observed that myelin and cell density gradually increased, which were positively and negatively correlated with FA and MD, respectively, across six layers in cortical regions. Similarly, ex vivo human tissue studies have demonstrated close associations between FA and myelin content (Schmierer et al. 2007) and between MD and cell density (Brabec et al. 2023). Also, the variation of FA in the cortical lamina is determined directly by the organizational structure of myelinated fibers (Leuze et al. 2014), and the cortical depth profiles of diffusivity reflect similar laminar structures correlated with cell density (Avram et al. 2022). Moreover, our findings are consistent with recent in vivo studies (Ali et al. 2022; J. Zhang et al. 2022), which revealed that both myeloarchitectonic and cytoarchitectonic structures influence diffusion properties in the cortex. We also observed that diffusional anisotropy increased and diffusivity decreased with layer increase in cortical regions, especially in prefrontal (IFC) and visual cortices. Notably, partial volume effects could contribute to the changes in cortical layer, particularly the pronounced increase in FA toward layer VI near the WM. Similar findings have reported a higher myelin content in deeper than superficial layers in the prefrontal cortex (Donahue et al. 2018; Miller et al. 2021) and hierarchically cytoarchitectonic features and myelin content in the visual cortex (Haenelt et al. 2023; Gomez et al. 2019). Collectively, our findings may indicate heterogeneous microstructural features in cortical layers of the human brain neocortex, and this provides complementary structural evidence supporting the existence of functional hierarchy.

In addition, it remains controversial whether the fixative has a significant effect (Richardson et al. 2014) or no effect (Shatil et al. 2018) on FA/MD values. Although each fixed hemisphere was washed with PBS for 2 days in this study, we cannot be certain that the fixative was fully washed for each case. Therefore, the potential influence of fixation on changes in layer microstructure needs further investigation, particularly in unfixed tissue.

### 4.2 Microstructure Changes in Different Cortical Layers of Aβ‐Positive Brains

Structural impairments in cortical layers have been reported in neurodegenerative diseases using various methods. For instance, cholesterol, an essential component of cell membranes, is unevenly distributed along cortical thickness and shows a layer‐dependent increase in layers III and IV but not in layers V and VI in AD cases (Lazar et al. 2013). An autoradiography binding study in postmortem human hemispheres found astrocytosis in superficial layers of the frontal cortex and all layers of the medial temporal gyrus and insular cortex in AD brains (Marutle et al. 2013). In a frontotemporal degeneration study based on ex vivo MRI, distinct upper layer hypointense bands and diffuse speckling on T2*‐weighted MRI were observed, corresponding to iron‐rich astrocytic processes and dystrophic patterns of microglia, respectively (Tisdall et al. 2022). These findings suggest that neurodegenerative diseases may selectively affect certain laminae of the cerebral cortex, associated with the cellular type comprising laminae.

The present study revealed an increasing trend in MD values in the Aβ‐positive group compared to the Aβ‐negative group in most layers, particularly in layers II/III. The MD reflects the overall contribution from different cellular components, and its increase is often associated with increased extracellular volume as a result of neural integrity collapse (Schaefer et al. 2000); for example, our histology‐dMRI correlation results in Figure S9 confirmed that increased MD is associated with decreased cell density. Since neurodegeneration often begins with loss of neural integrity and ultimately results in cell death, the MD may show high sensitivity to early signs of neurodegeneration (Tripathi 2012). Therefore, increased MD values in Aβ‐positive cases in this study may reflect an early alteration of cytoarchitecture in cortical layers of the neocortex. Note that in our study, the Aβ‐positive subjects were in the predromal stage and had not reached the diagnosis of AD; thereby, the change of layer MD in these samples indicated its sensitivity for early detection.

Additionally, this study found a significant positive correlation between MD values in layer IV of the IFC and Amyloid stage but not in other layers, highlighting the layer‐specific effect of Aβ pathology on cortical microstructure. The frontal lobe is one of the main areas of Aβ deposition and initially develops in the orbital frontal cortex (Fouquet et al. 2014). APOE ε4 carriers exhibit abnormal hyperintensities on FLAIR images in the IFC compared to non‐APOE ε4 carriers, possibly reflecting aberrant Aβ accumulation and excretion (Zhang et al. 2021). DTI studies have reported that AD is associated with lower FA and higher MD in the frontal lobe, suggesting its key role in AD progression (Matthews et al. 2013; Sexton et al. 2011). Thus, the positive correlation between MD and Amyloid stage found in the present study indicates a close relationship between microstructural damage in the IFC and Aβ deposition, and the MD value in layer IV of the IFC may serve as a biomarker for AD staging.

### 4.3 Cortical Layer‐Dependent and Region‐Specific Distribution of AD Pathology

A previous postmortem study using molecular imaging tracers found a clear lamination pattern with high fibrillar Aβ in the frontal cortex but low fibrillar Aβ in the medial temporal gyrus and insular cortex in AD cases (Marutle et al. 2013). A recent ex vivo human brain study reported widespread Aβ deposition across cortical layers in the anterior temporal and orbitofrontal cortex in AD, with layers IV–VI exhibiting larger plaques and more accumulation than layers II–III (Tisdall et al. 2022). Tau accumulation in AD also showed layer dependence, with layers II–III and V of the frontotemporal cortex (Bussière et al. 2003; Hof et al. 1990) and layer II of the entorhinal cortex (Gómez‐Isla et al. 1996) being vulnerable to the laminar distribution of NFTs. Similarly, our study observed layer‐dependent nonlinear changes from the pial to WM surface for Aβ deposition in the frontal and parietal cortex of AD samples and for Tau deposition in the temporal cortex of AD and PART samples. This may indicate that Aβ and Tau protein distribution is layer‐dependent and region‐specific in the neocortex.

### 4.4 Correlation Between Cortical Microstructure and AD Pathology

In AD mice with tau pathology, a positive correlation was observed between neurite density index from dMRI model in the cortex and levels of tau protein (Colgan et al. 2016). In 3xTg AD mice with both Aβ plaques and neurofibrillary tangles, abnormal DTI measurements were found in GM regions where both congophilic Aβ plaques and hyperphosphorylated tau accumulation were present (Snow et al. 2017). In transgenic 5xFAD mice, layer‐specific enriched Aβ disrupted cross‐laminar processing of columnar microcircuits, impairing the integrity of cortical columns (Lison et al. 2014). A recent ex vivo human brain study in AD using T2‐weighted MRI found significant relationships between phosphorylated tau and cortical thickness in primary visual cortices, temporal pole, insular cortex, and posterior cingulate gyri (Sadaghiani et al. 2022). Our previous ex vivo studies in the human hippocampus revealed significant correlations between magnetic susceptibility and Aβ/Tau density in AD and PART, as well as between dMRI measurements and Aβ/Tau density in AD (Zhao et al. 2022, 2021). These findings indicate that changes in MRI could reflect the effects of Aβ or Tau or both on the brain. Consistently, our current dMRI‐histology analysis found that ΔMD (difference between age‐ and sex‐matched AD and HC) showed significant positive correlations with Aβ density but not Tau density across six cortical layers in the frontal and parietal cortices. Combining with our finding abovementioned that MD was negatively correlated with cell density in Figure S9, this result may reflect the specific effect of Aβ‐pathology on the cellular microstructure of cortical layers. Similar findings have been reported in in vivo studies, with higher MD in cortical regions of AD brains associated with higher pTau/Aβ42 in cerebrospinal fluid (Hoy et al. 2017). Recent dMRI‐PET in vivo studies have demonstrated a significant correlation between Aβ/Tau burden and cortical microstructure in AD brains (Nakaya et al. 2022; Prescott et al. 2022; Spotorno et al. 2023). Both the direct evidence from ex vivo histology‐dMRI correlation analysis and indirect evidence from in vivo studies collectively support the use of dMRI‐based microstructural markers for non‐invasive characterization of the spatial distribution of AD pathology.

### 4.5 Limitations

There were several limitations to our study. First, our sample size was too small for quantitative group comparison and correlation analysis, which is typical for ex vivo human brain studies. The findings in changes of cortical layer microstructures in early AD and the relationship between diffusivity and Aβ/Tau burden need to be verified through large‐sample studies in the future. Second, the cortex thickness is approximately 3 mm, while the current dMRI resolution is 0.8 mm isotropic. Although we interpolated dMRI to high‐resolution T2‐weighted images at 0.5 mm isotropic resolution, partial volume effects may be inevitable. Thus, future studies using dMRI at higher resolution are needed to verify our findings. Third, inadequate washing of the fixed hemisphere could potentially result in higher water density in outer layers compared to inner layers, potentially influencing diffusivity variations across cortical layers. Fourth, the histological images were not co‐registered to the ex vivo MRI scans in this study. Instead, a qualitative registration was performed based on prior anatomical knowledge from corresponding areas within the same brain. Future studies may need to verify the relationship between the layer‐dependent microstructural profiles using advanced registration methods, such as the Tensor Image Registration Library (Huszar et al. 2023). Finally, several alternative methods, such as AI‐based techniques, may offer more accurate measures of cell, Aβ, and Tau densities compared to color deconvolution and should be considered for verifying our findings in future studies.

## 5 Conclusion

In summary, the present study used high‐resolution dMRI in ex vivo human hemispheres to reveal a layer‐dependent pattern in cortical microstructure and observe Aβ‐related diffusivity changes in selected cortical layers and regions. Combined with histological data, we found that increases in diffusivity were positively correlated with Aβ but not Tau deposition across cortical layers. These findings provide direct evidence that Aβ‐pathology has a significant effect on the dMRI‐based microstructure of the neocortex, which can be potentially used as early marker of AD pathology.

## Conflicts of Interest

The authors declare no conflicts of interest.

## Supporting information


**Data S1.**hbm70222‐sup‐0001‐Supinfo.

## Data Availability

The data that support the findings of this study are available from the corresponding author upon request.
