# Supplementary material for: Layer‐Dependent Effect of Aβ‐Pathology on Cortical Microstructure With Ex Vivo Human Brain Diffusion MRI at 7 Tesla
Source: Hum Brain Mapp. 2025 May 2;46(7):e70222. doi: 10.1002/hbm.70222 (PMC12046383; doi:10.1002/hbm.70222)
Supplement: Supplementary file 1 — Data S1.hbm70222‐sup‐0001‐Supinfo. [file HBM-46-e70222-s001.docx]

**Layer-dependent effect of Aβ-pathology on cortical microstructure with ex-vivo human brain diffusion MRI at 7 Tesla**

**Supplementary Methods**

**Immunohistochemistry**

The brain tissue sections were dewaxed in xylene, rehydrated through the decreasing concentration series of ethanol, and washed in 1× phosphate-buffered saline (PBS). Then, slices were blocked with 3% H_2_O_2_ for 10 min and immerged in boiling citrate buffer (pH 6.0) for 15 mins, and washed twice in PBS. The slices were immunolabeled with the primary antibodies (AT8 or Aβ as listed in Table S1) that were diluted in blocking buffer (1× PBS+0.1% Triton +3% normal goat serum) and incubated at 4°C overnight. After being transported to 37 °C for 60 mins, slices were treated with a secondary antibody (goat anti-mouse IgG) at 37 °C for 60 mins and washed in 1× PBS. The slices were immersed with 3, 3’-diaminobenzidin (DAB), kept at room temperature for 3 mins. Finally, the sections were dehydrated, cleared and mounted with neutral gums.

**Table S1 Primary antibodies used for immunohistochemistry staining.**

| **Primary Antibodies** | **Abbreviation** | **Host** | **Clone** | **Dilution** | **Vendor** |
| --- | --- | --- | --- | --- | --- |
| Anti-Hyperphosphorylated-tau | AT8 | Mouse | MN1020 | 1:200 | Thermo Fisher Scientific |
| Anti-amyloid β protein | Aβ | Mouse | A3981 | 1:200 | Sigma-Aldrich |

**Gray and White Matter Segmentation**

nnU-Net is an open-source deep learning framework that has been specifically designed for medical image segmentation (Isensee et al., 2021), which is based on an encoder-decoder design to learn hierarchical representations of input images and generate corresponding segmentation maps (Zhou et al., 2019;Çiçek et al., 2016). In this study, we labeled a subset of slices along the sagittal axis to train our model using the 2D approach. Specifically, for each *ex-vivo* hemisphere, in the same locations, we manually labeled gray and white matter (GM/WM) on five slices per brain with a 3 mm interval between adjacent slices. Then, we used fifty slices from ten of the fifteen hemispheres (four Aβ-negative cases and six Aβ-positive cases) for training, and twenty-five slices from the remaining five samples as the test dataset. The 2D U-Net was trained using PyTorch 1.8.1 on Nvidia GeForce RTX 3090, with a batch size of 8, a patch size of 448×256, depth of 7, and a base channel number of 32. The model was optimized using a loss function based on dice coefficient (DC) and cross-entropy (CE), and five-fold cross-validation was employed during training. The test results showed that our trained model had a high predicting accuracy with an averaged DC > 0.8 in five samples. Finally, the trained model was used to segment all slices of all samples to generate the cortical GM and WM labels.

**Supplementary Results**

**
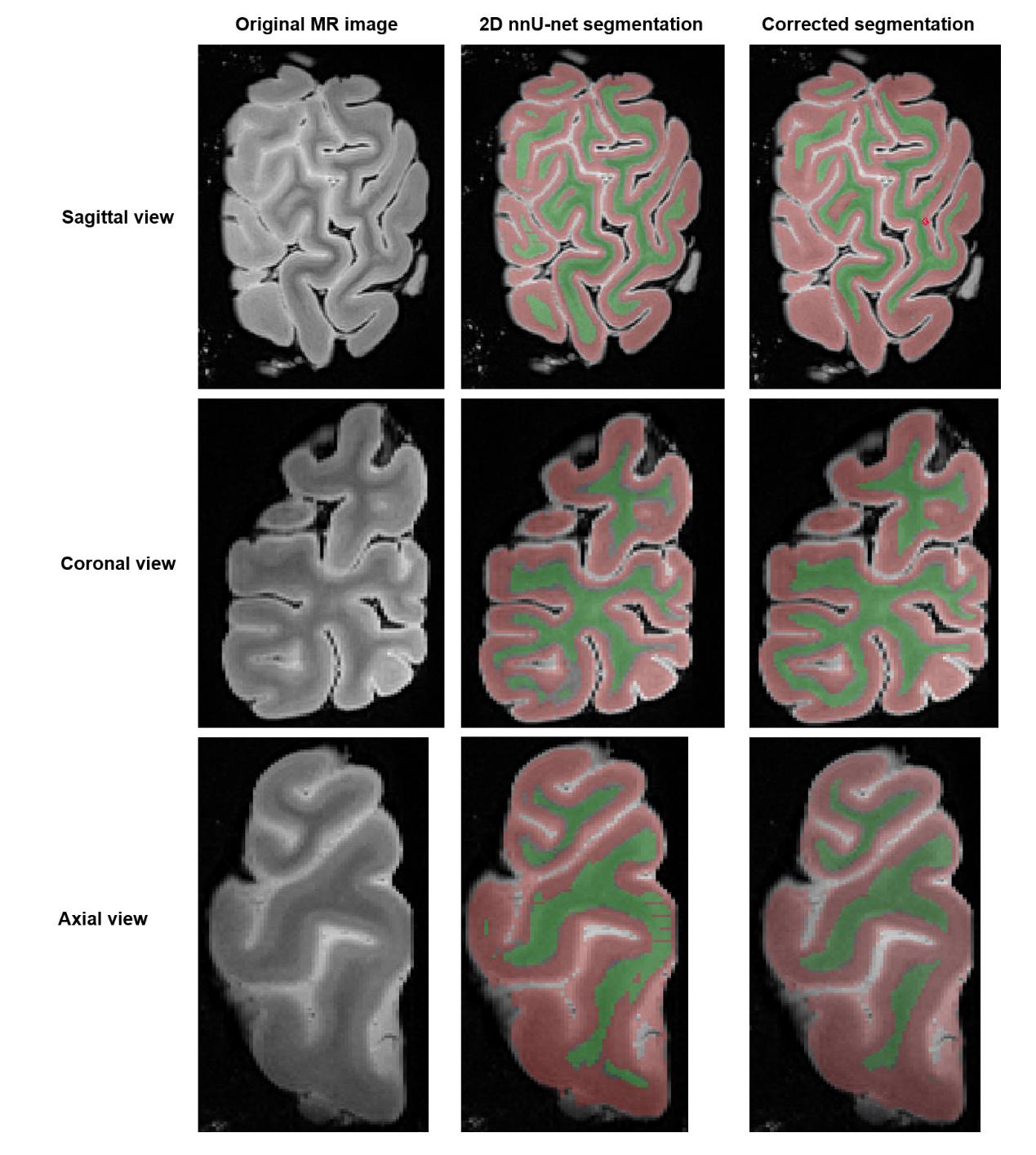
Figure S1 The display of GM-WM segmentations of 2D nnU-net and manual corrections in three slices with little tissue in one case.**


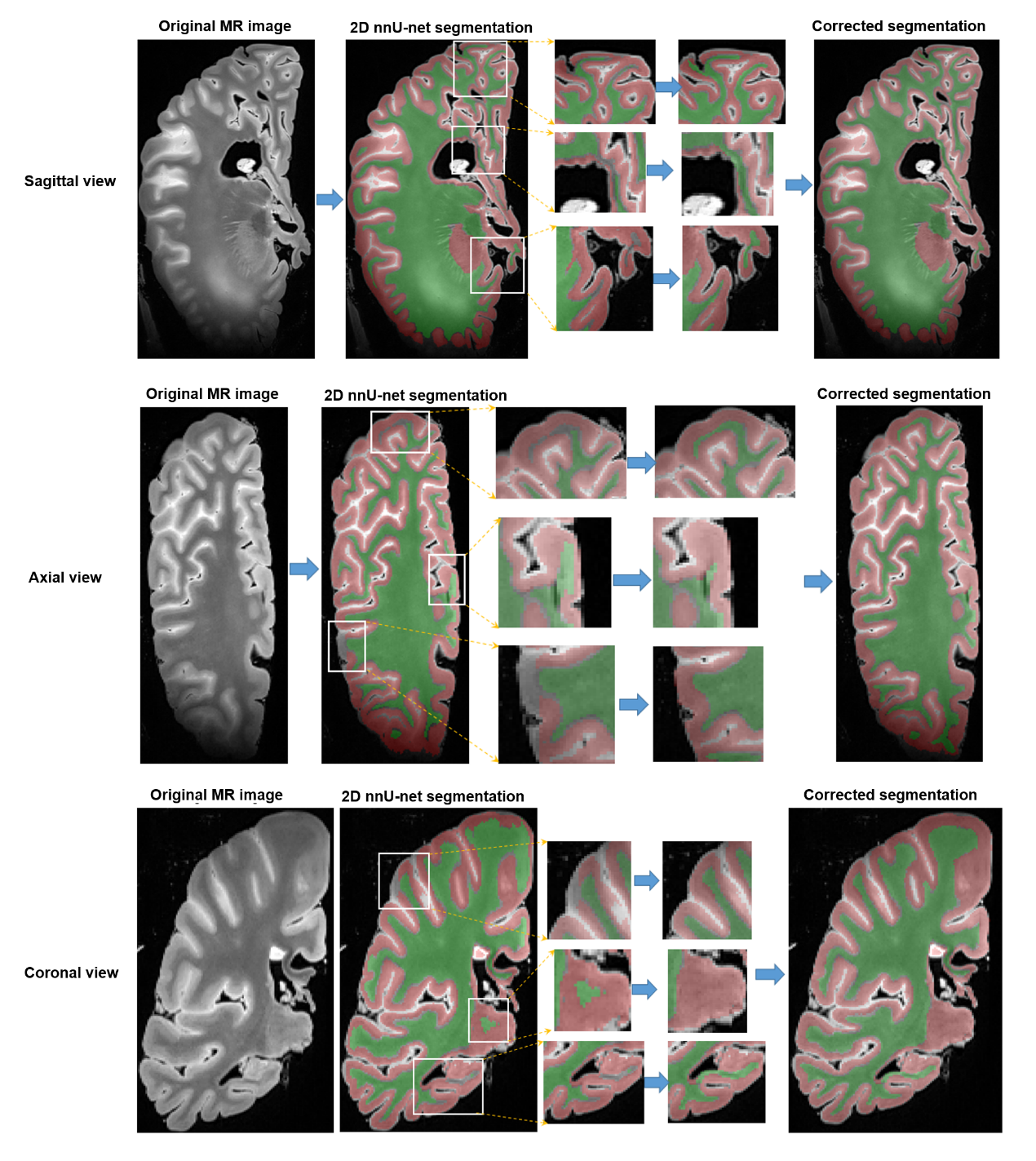


**Figure S2 The display of GM-WM segmentations of 2D nnU-net and manual corrections for regional boundary areas in three slices of one case** **across sagittal, coronal and axial views.**


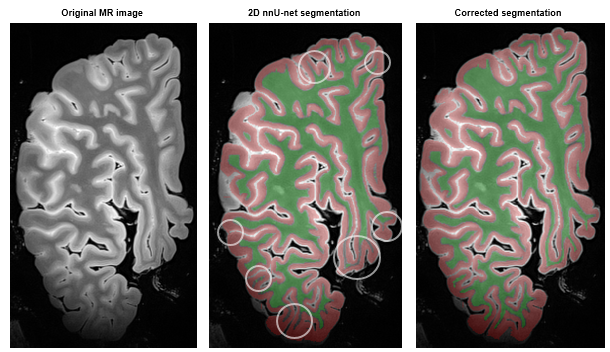


**Figure S3 The display of GM-WM segmentations of 2D nnU-net and manual corrections for the areas with buried sulcus in one slice of one case.**


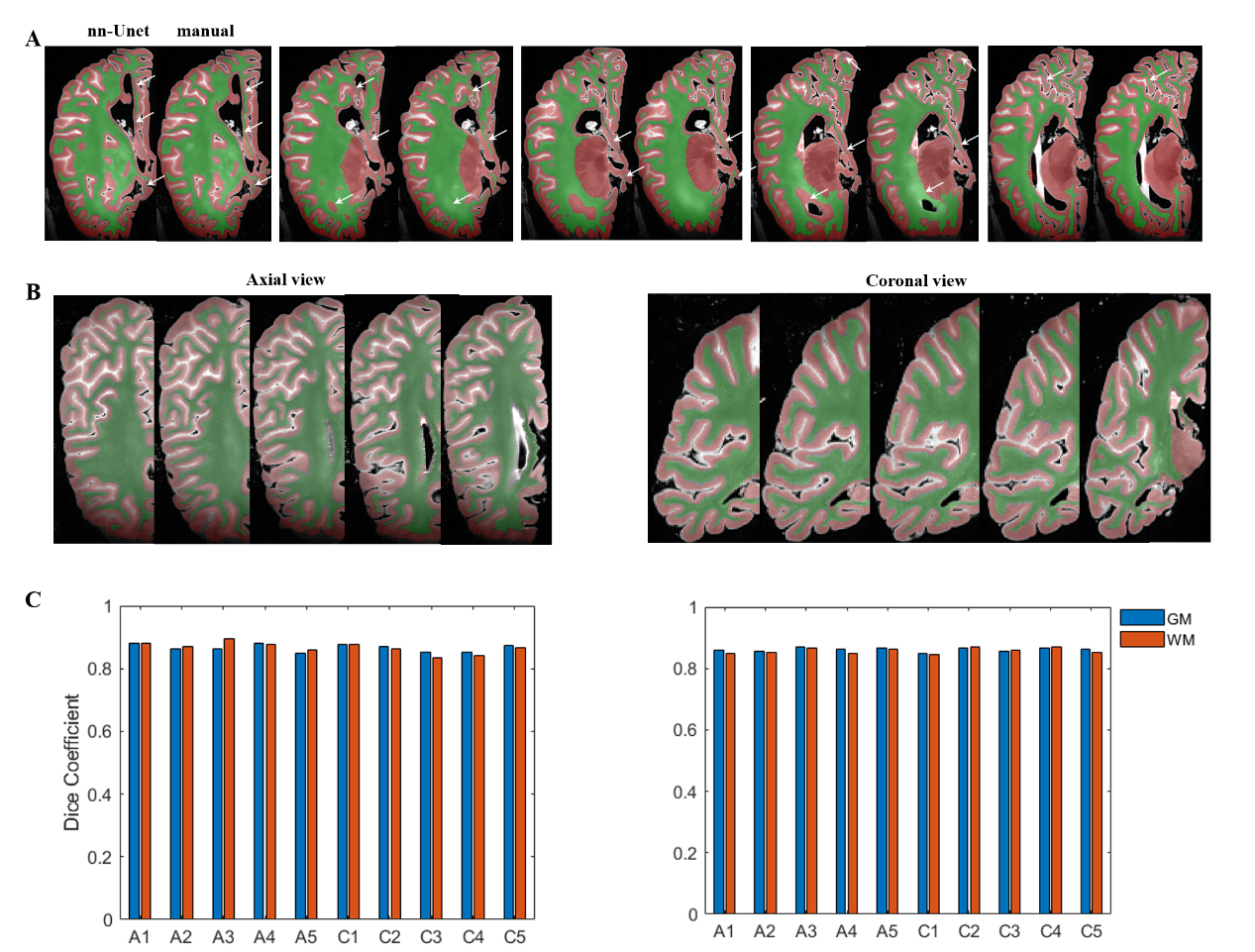


**Figure S4 Comparisons between nnU-net and manual segmentations in five slices from two samples.** Panel A shows five paired slices with nn-Unet and manual segmentations from PART1 along the sagittal axis, with minor differences observed in a few regions, highlighted by white arrows. Panel B shows five slices from the nn-Unet segmentations after manual correction in PART1, viewed along the axial and coronal axes. Panel C shows slices A1-A5 and C1-C5 from PART1 (left) and AD3 (right), respectively, across axial and coronal views, while the network was trained using sagittal sections. GM: grey matter; WM: white matter.

**Figure S5 Comparison of Thickness between Our Results and HCP.** (A) shows the thickness differences (< 0.5mm) between our results and HCP in 112 brain regions of four HC cases. (B) shows significant positive correlations between our results and HCP (Reference Data) across the
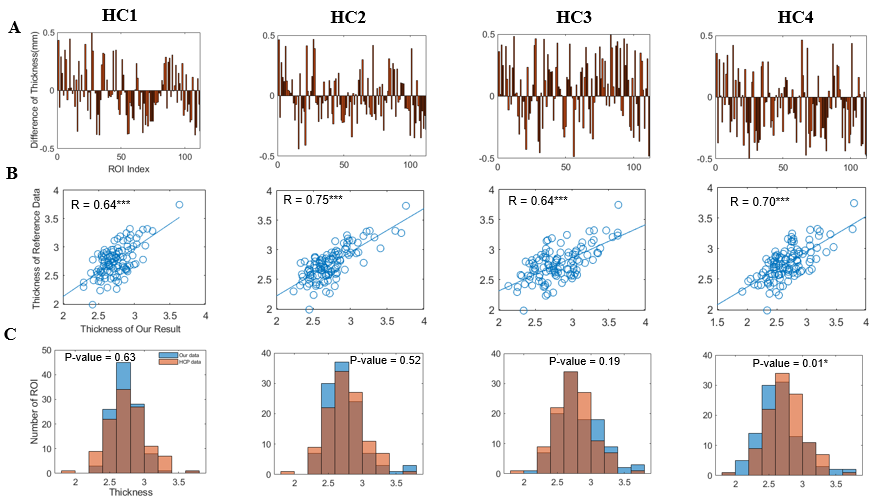
112 regions. (C) shows that, using a two-sample Kolmogorov-Smirnov test, the thickness of the 112 regions in our results did not significantly differ from those in HCP for all HC cases except for case 4. The findings in (B) and (C) suggest a commonly reliable registration in selected ROIs for four HC samples. ***: *P* < 0.001.


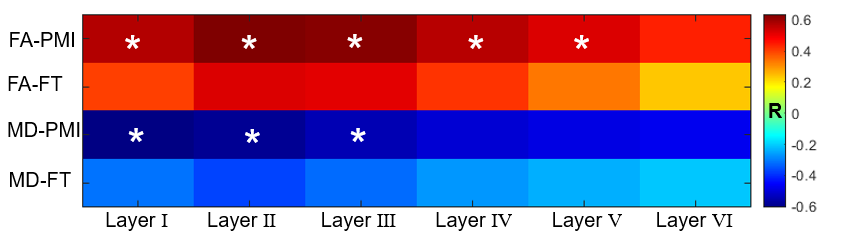


**Figure S6 Correlations between PMI/FT and FA/MD across all samples.** PMI: post-mortem interval; FT: fixation time; FA: fractional anisotropy; MD: mean diffusivity. *: 0.01 < p < 0.05.


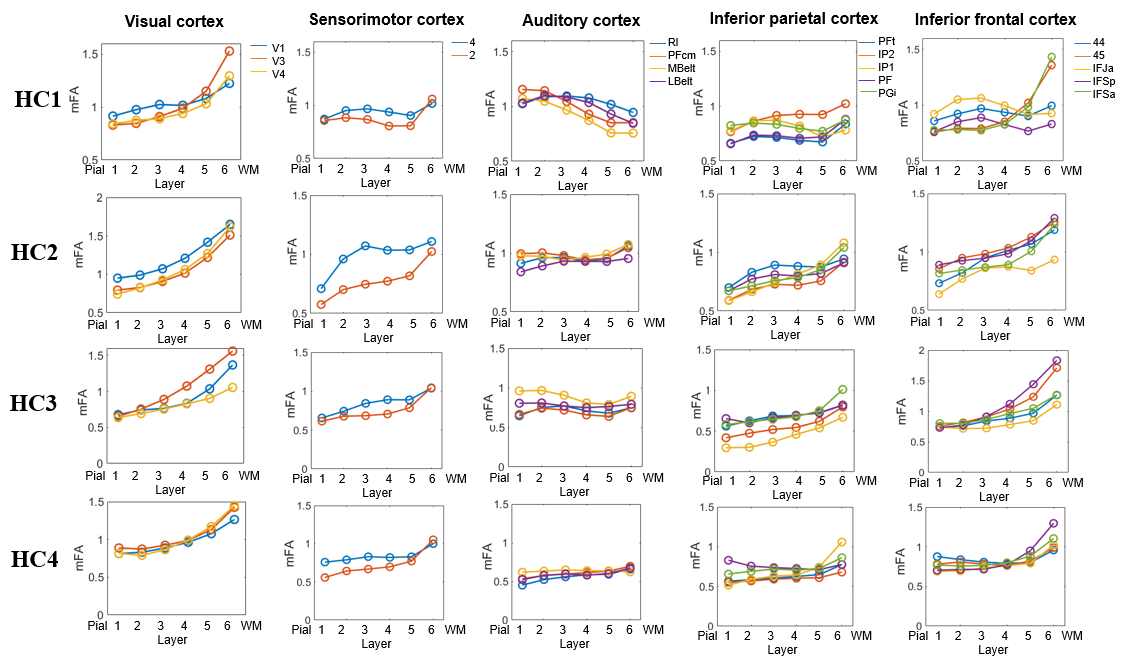
**Figure S7 Laminar-specific fractional anisotropy (FA, normalized) in different cortical regions of four HC cases**


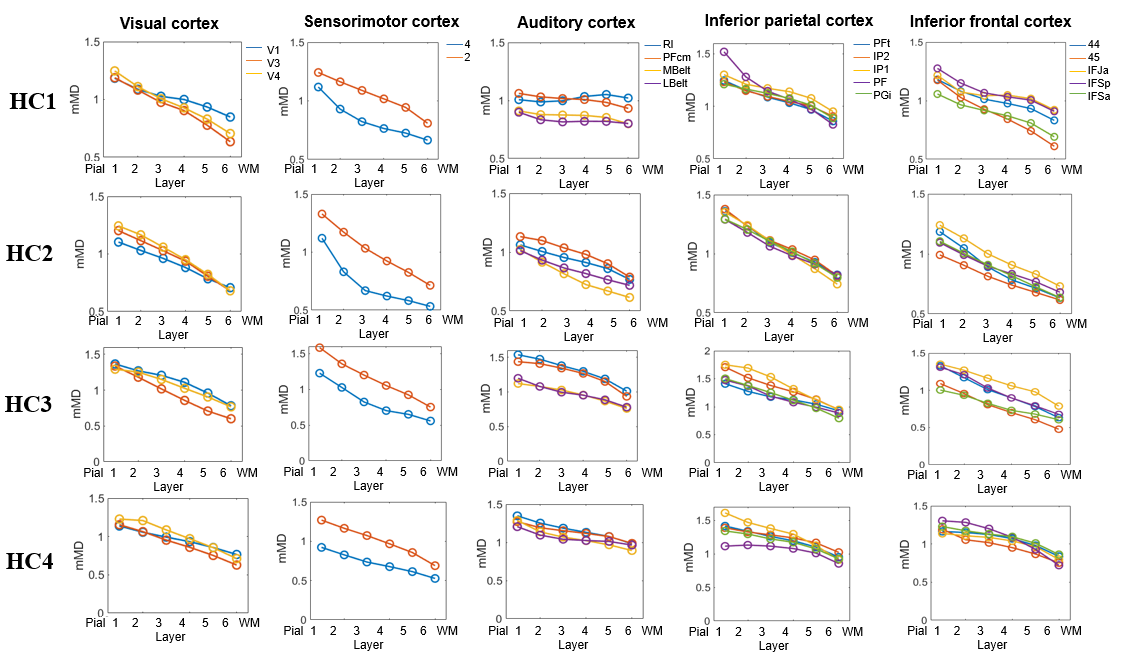


**Figure S8 Laminar-specific mean diffusivity (MD, normalized) in different cortical regions of four HC cases.** Note that the axial and radial diffusivity showed similar patterns with mean diffusivity, and therefore are not displayed here


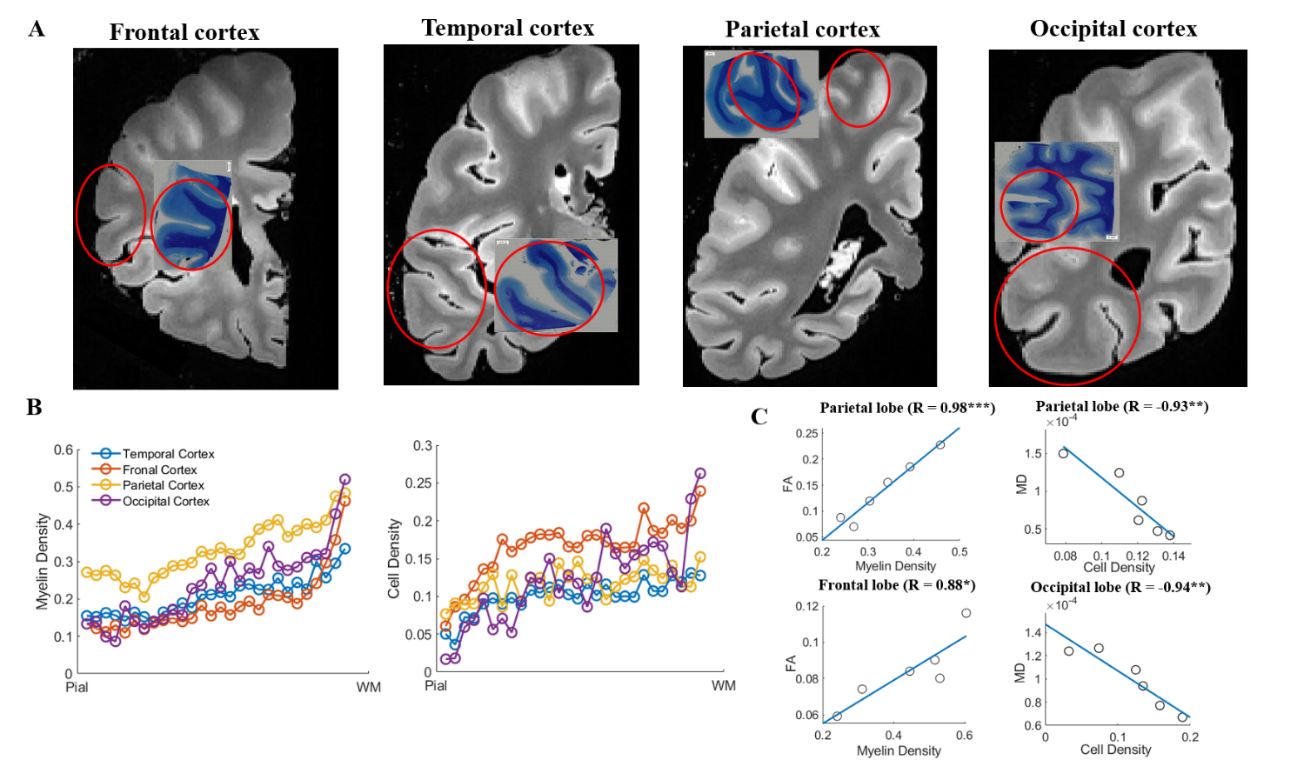
**Figure S9 Relationships between dMRI measurements and cell/myelin density.** (A) shows manual matching of MRI sections with histological images. The regions within red circles in the MRI are most similar to histological images. (B) displays changes in myelin density based on Klüver-Barrera staining and H&E-based cell density with laminar depth from the pial to WM surface in four cortical regions. Note here the cortex in the histological images are divided into 30 layers for more detailed depiction. (C) shows significant correlations between FA and myelin density and between MD and cell density in several cortical regions. *: 0.01 < *P* < 0.05; **: 0.001 < *P* < 0.01; ***: *P* < 0.001


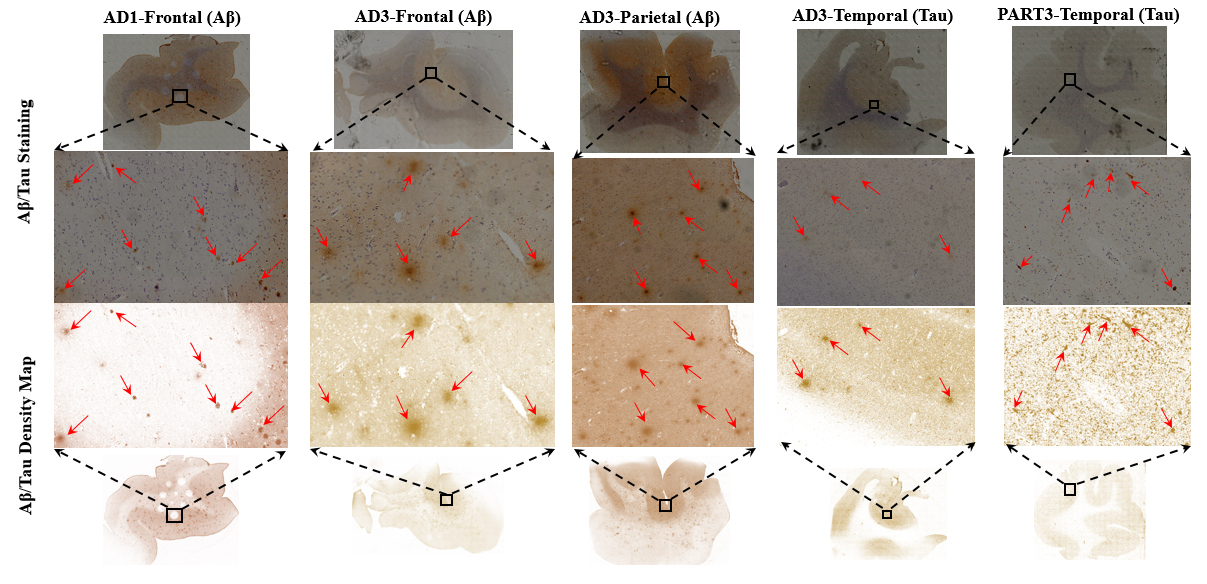


**Figure S10 Aβ/Tau plaque deposotion in two Aβ-positive and one PART samples.** The top panel shows original staining images and the bottom panel shows corresponding Aβ/Tau density maps obtained after color deconvolution and their local close-up images. Aβ or Tau plaques are highlighted with red arrows.

**Reference**

Isensee, F., Jaeger, P. F., Kohl, S. A., Petersen, J., & Maier-Hein, K. H. (2021). nnU-

Net: a self-configuring method for deep learning-based biomedical image segmentation.

Nature methods, 18(2), 203-211.

Zhou, Z., Siddiquee, M. M. R., Tajbakhsh, N., & Liang, J. (2019). UNet++: A Nested U-Net Architecture for Medical Image Segmentation. In Deep Learning in Medical Image Analysis and Multimodal Learning for Clinical Decision Support (pp. 3-11). Springer.

Çiçek, Ö., Abdulkadir, A., Lienkamp, S. S., Brox, T., & Ronneberger, O. (2016). 3D U-net: learning dense volumetric segmentation from sparse annotation. In International conference on medical image computing and computer-assisted intervention (pp. 424-432). Springer.
